# Bilateral lung transplantation after caesarean section in pregnancy with severe pulmonary arterial hypertension: A case report

**DOI:** 10.1097/MD.0000000000018109

**Published:** 2019-11-22

**Authors:** Jia Ye, Jing-Yu Chen, Na Xu, Bo Wu, Zhi-Ping Wang, Hong-Yang Xu, Jin-Qi Ma

**Affiliations:** a Department of Obstetrics and Gynecology; b Department of General Thoracic Surgery, Lung Transplantation Group; c Department of Anesthesiology; d Department of Critical Care Medicine, The Affiliated Wuxi People's Hospital of Nanjing Medical University, Wuxi, Jiangsu, China.

**Keywords:** atrial septal defect, caesarean section, extracorporeal membrane oxygenation, lung transplantation, pulmonary arterial hypertension

## Abstract

**Rationale:**

Pulmonary arterial hypertension (PAH) can lead to an increase in right ventricular load and subsequently heart failure, making severe PAH a contraindication for pregnancy. In addition, PAH may worsen during pregnancy and puerperium, which requires high-quality critical care. This report is the first instance in which a patient with severe PAH, survived a successful atrial septal defect (ASD) repair and bilateral lung transplantation during puerperium.

**Patient concerns:**

A 42-year-old pregnant woman with congenital heart disease (CHD) and severe PAH was admitted to our hospital for the management of pregnancy and delivery. The patient was diagnosed with severe PAH in 2013, and no significant improvements or deteriorations were found until this pregnancy-related hospital admission.

**Diagnosis:**

The patient was diagnosed with CHD and severe PAH in 2013 with color Doppler echocardiography, right cardiac catheterization, and pulmonary perfusion imaging. The patient's mean pulmonary arterial pressure increased to 140 mm Hg during pregnancy, suggesting an exacerbated PAH with high risks to both her and the unborn child.

**Interventions:**

The patient was treated with PAH-targeting treprostinil injection to reduce pulmonary artery pressure. Caesarean section was performed at 27 weeks and 5 days of gestation. The patient was put under extracorporeal membrane oxygenation (ECMO) with the help of local anesthesia before the operation. The investigators finally conducted a bilateral lung transplantation with a shell incision of the sternum under cardiopulmonary bypass.

**Outcomes:**

The mother and the neonate survived and recovered well after the operation, and were discharged from the hospital on the fourth month post-hospitalization.

**Lessons:**

Severe PAH is an absolute contraindication for pregnancy. However, for patients who insist on a pregnancy, it could be plausible to proceed with a targeted drug therapy and ECMO after conducting a cesarean section, and finally, a lung transplantation. Multidisciplinary diagnosis and treatment is the key to the successful treatment of a PAH-complicated pregnancy.

## 1 Introduction

Pulmonary arterial hypertension (PAH) is a pathophysiological syndrome characterized by pulmonary vascular remodeling, pulmonary arterial pressure, and pulmonary vascular resistance.^[1]^ Severe PAH is a contraindication for pregnancy. However, several patients with PAH are eager to get pregnant and have children. Determining methods to improve the pregnancy outcomes is a challenge for obstetricians. A case of pregnancy, complicated with congenital heart disease (CHD) and severe PAH, was treated successfully by a bilateral lung transplantation after caesarean section. According to the present literature, this is the first patient to successfully undergo an atrial septal defect (ASD) repair along with a bilateral lung transplantation during puerperium.

The 2018 European Society of Cardiology (ESC) guidelines classify such patients as “extremely high risk of maternal mortality or severe morbidity,” which is a contraindication for pregnancy, and should be treated in a tertiary critical care center for pregnancy and cardiac diseases.^[2]^

## 2 Case presentation

A 42-year-old pregnant woman with CHD and severe PAH was admitted to our hospital for the management of pregnancy and delivery. The patient was diagnosed with CHD and severe PAH in 2013 by color Doppler echocardiography, right cardiac catheterization, and pulmonary perfusion imaging. Color Doppler echocardiography showed that the patient suffered from CHD with a left to right shunt and an ostium secundum type of ASD. A right cardiac catheterization revealed that the mean pulmonary arterial pressure (mPAP) was 104 mm Hg while pulmonary perfusion imaging revealed a bilateral pulmonary perfusion injury. No significant improvements or deteriorations were detected in the patient's medical condition before the current pregnancy. There was no history of CHD or PAH in her family and patient had a healthy lifestyle.

Patient was admitted to the pulmonary vascular department at 21 weeks and 3 days of gestation. The complete process of diagnosis and treatment is presented in Table 1.

**Table 1:**
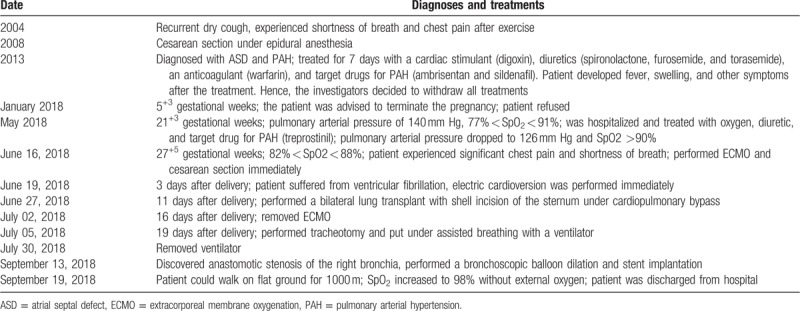
Overview of diagnoses and treatments.

The patient was first treated with a cardiac stimulant (digoxin), diuretics (spironolactone, furosemide, and torasemide), an anticoagulant (warfarin), and targeted drugs (ambrisentan and sildenafil) in 2013 after being diagnosed with ASD and PAH. However, after 7 days of treatment, patient developed fever, swelling, and other symptoms. Hence, the investigators decided to withdraw all treatments. After 3 years, the symptoms did not worsen significantly. Reexaminations repeatedly showed that the mPAP was approximately 110 mm Hg, while the cardiopulmonary exercise test revealed a moderate limitation in activity. The patient previously had 1 caesarean section 12 years ago when she was not diagnosed with PAH, in addition to a history of 5 induced abortions. The patient had cough, chest tightness, shortness of breath, and recurrent epistaxis after 5 months of gestation. Her mPAP at this stage had increased to 140 mm Hg. The patient and her family were informed of the maternal, fetal, and neonatal risks due to exacerbated PAH. However, they decided to continue with her pregnancy. Patient was then admitted to our hospital at 21 weeks and 3 days of gestation.

The patient's pulse, blood pressure, body temperature, respiratory rate, and oxygen saturation on admission were 104 beats/minute, 121/64 mm Hg, 37.2°C, 16 breaths/minute, and 77% to 91%, respectively. A physical examination showed that the patient had slight cyanosis of the lips, clubbed fingers and toes, a second cardiac sound of pulmonary artery hyperactivity, an audible second intercostal of left thoracic duct, and a systolic murmur of grade II. Laboratory examinations revealed an increased brain natriuretic peptide level of 159 pg/mL. A pulmonary computed tomography revealed no obvious signs of pulmonary embolism.

After admission, oxygen inhalation and PAH-targeting treprostinil injection were used to reduce pulmonary artery pressure. Initially, the dose of treprostinil was 1.25 ng/kg/minute which was gradually increased to 8.75 ng/kg/minute with a subcutaneous micropump, resulting in an increase in resting pulse oxygen saturation (SpO_2_) from 77% to 90%. At the 26th week of gestation, the fetal body mass was estimated to be 1000 g. Five milligrams of dexamethasone was injected 4 times, every 12 hours, for a total dose of 20 mg, to accelerate the fetal lung maturation. A reexamination using a color Doppler echocardiography revealed that mPAP was 126 mm Hg after the treatment.

At 27 weeks and 5 days of gestation, the patient condition worsened. Patient had an obvious chest tightness and shortness of breath, and the SpO_2_ could only be maintained at 82% to 88% under continuous high flow of oxygen inhalation (35 L/min, 95% oxygen concentration). Hence, an emergency operation was conducted. The patient was placed under an extracorporeal membrane oxygenation (ECMO) under local anesthesia before the operation. Then, a caesarean section, bilateral ascending uterine artery ligation, bilateral ovarian artery communicating branch ligation, and bilateral oviduct ligation were performed under general anesthesia. A baby boy weighing 1150 g was delivered successfully. The Apgar scores were 8, 9, and 7 at 1, 5, and 10 minutes, respectively, and the baby was placed under intensive care. Uterine contraction was promoted by pouring 20 units of oxytocin after amputating the umbilical cord. The total bleeding volume was 250 mL during the operation. The vital signs were found to be stable after the operation.

The patient was given cardiopulmonary support, in addition to cefoxitin to prevent infection, ambroxol to reduce phlegm, esomeprazole for alkalization, treprostinil to control PAH, and diuretics to improve cardiac function and maintain a stable internal environment in the intensive care unit. A total of 170 mL of lochia was excreted within 24 hours postpartum, and the uterus contracted well. After the treatment, however, it was difficult to improve the cardiopulmonary function of the patient. Eleven days postpartum, the patient underwent “repair of atrial septal defect (ASD) and bilateral lung transplantation with shell incision under cardiopulmonary bypass.” After the operation, patient was put under ventilator-assisted respiration and ECMO support. Treatments with antibiotics, noradrenaline pressor; sedation with midazolam; analgesia with remifentanil; diuresis with furosemide; immunosuppression with cyclosporine and prednisolone were performed methodically. Human serum albumin was also given for improved recovery. ECMO was successfully removed 16 days after delivery. The patient was discharged 86 days after cesarean section.

The premature infant was treated with ventilator-assisted breathing, antibiotic treatment, pulmonary surfactant replacement, and phototherapy in the neonatal intensive care unit. A color Doppler echocardiography revealed a patent ductus arteriosus of the infant, an ostium secundum defect of 2 mm, and a small amount of left to right shunt. The infant was discharged 55 days after birth, weighed 2140 g, and had feeding tolerance, and no neurological abnormalities were seen.

## 3 Discussion

In normal subjects, mPAP at rest is 14 ± 3 mm Hg (1 mm Hg = 0.133 kPa). An mPAP of ≥25 mm Hg measured by right cardiac catheterization, a pulmonary artery wedge pressure of ≤15 mm Hg, and a pulmonary vascular resistance of >3 wood units is diagnosed clinically as PAH.^[3]^ The clinical causes for PAH include idiopathic, hereditary, drug, and toxin induction, connective tissue disease, HIV infection, portal hypertension, CHD, schistosomiasis, etc. Based on mPAP levels, PAH is classified as mild (30–50 mm Hg), light (50–80 mm Hg), and severe (>80 mm Hg).^[4]^

The main symptoms of PAH are pain and tightness in the chest, palpitations, shortness of breath, dyspnea, hemoptysis, and swelling in the lower extremities. Physical examinations in PAH patients should be focused on cardiac auscultation. Laboratory tests are recommended for routine biochemical, immunological, HIV, and infection tests, in order to identify high-risk factors. The first evaluation method of choice for monitoring the state of pregnancy is color Doppler echocardiography.^[5]^ Right cardiac catheterization is the gold standard for diagnosing PAH, but it is rarely used in pregnant women due to its invasiveness. Hemodynamic tests should be carried out after delivery to determine the etiology of PAH.

The maternal and fetal mortality rates in pregnant women with severe PAH are approximately 30% to 56% and 11% to 28%, respectively.^[6]^ The 2018 ESC guidelines do not recommend pregnancy in patients with PAH. Patients with PAH should be informed of possible life-threatening worsening of health during pregnancy and puerperium. Patients with severe PAH and/or cardiac function of grade III should be advised to undergo therapeutic abortion before 22 weeks of gestation.^[7]^

The treatments used to manage women with PAH, who insist on pregnancy, are as follows^[8]^: recommending their treatment and medical follow-up to PAH treatment centers that comprise of PAH specialists, obstetricians, anesthetists, and critical care doctors; immediately terminating the use of endothelin receptor antagonists due to their teratogenic effects; starting or continuing the use of phosphodiesterase type 5 inhibitor and/or prostacyclin B; applying low-molecular-weight heparin for anticoagulation, long-term oxygenation, and diuretics; following-up monthly by a color Doppler echocardiography to assess and treat right ventricular decompensation and right heart failure; and performing caesarean section at 32 to 36 weeks of gestation under spinal-epidural anesthesia with due consideration to the patient's medical condition.

The risk of caesarean section in PAH patient is extremely high. Complications such as acute right heart failure, pulmonary hypertension crisis, or respiratory failure may occur during and after caesarean section. The perioperative period during a cesarean section should be well evaluated, and prevention and treatment plans for complications, if any, should be prepared. Putting the patient under ECMO before caesarean section is one of the methods to reduce risks during the operation. ECMO can enhance oxygenation, improve hypoxic condition, and provide respiratory and circulatory support, thus, increasing patient tolerance to the surgery. ECMO can also avoid the shortcomings of a conventional cardiopulmonary bypass and simplify perioperative airway management.^[8]^ In order to control postpartum hemorrhage, uterine devascularization and bilateral tubal ligation may be performed.

Maternal and neonatal care is very important following delivery. The highest maternal mortality rate has been found within 4 weeks postpartum. The main cause of death is mostly a right ventricular failure, while other causes include an excessive increase in pulmonary vascular resistance and thromboembolic events.^[3]^ Postpartum treatment for reducing the risk of right ventricular failure include nitric oxide inhalation and the usage of cycloprostin, iloprost, and vasopressin. The prevention of postpartum hemorrhage is crucial and oxytocin remains the first-line drug, but it must be used with utmost care, since it can lead to systemic hypotension and reflex tachycardia, which may cause an increase in the pulmonary arterial pressure.^[7]^ Women with PAH are generally not recommended to breastfeed after delivery. Regular follow-up visits are also required puerperium. If the postpartum condition does not improve, lung transplantation is the ultimate life-saving option before an irreversible end-stage organ damage. The long-term patient survival rate following a cardiopulmonary transplant is lower than that of a bilateral lung transplant.^[9]^ Therefore, bilateral lung transplant should be first considered for PAH patients who need transplantation.^[8]^

## 4 Conclusion

Following the successful treatment in the present case, multidisciplinary diagnosis and treatment model can be considered key for the successful treatment of pregnancy with PAH. ECMO support is recommended during a caesarean section in such patients. Performing a uterine devascularization during surgery can significantly reduce postpartum hemorrhage. A comprehensive evaluation for possible lung transplantation for critical cases should be carried out in time. Lung transplantation is an effective treatment in critical conditions.

## Author contributions

**Conceptualization:** Jia Ye.

**Data curation:** Jia Ye, Bo Wu, Zhi-Ping Wang, Hong-Yang Xu.

**Funding acquisition:** Jin-Qi Ma.

**Resources:** Jia Ye, Na Xu.

**Supervision:** Jing-Yu Chen, Jin-Qi Ma.

**Writing - Original Draft:** Jia Ye, Jin-Qi Ma.

**Writing - Review & Editing:** Jing-Yu Chen, Jin-Qi Ma.
